# OUTCOMES FOR AN INTERGENERATIONAL TECHNOLOGY PROGRAM: A PRE–POST WITH WAITLIST CONTROL STUDY

**DOI:** 10.1093/geroni/igae098.0704

**Published:** 2024-12-31

**Authors:** Meaghan Colvin, Skye Leedahl, Cindy Tsotsoros, Melanie Brasher, Gina Barbera, Josie Santilli

**Affiliations:** University of Rhode Island, Kingston, Rhode Island, United States; University of Rhode Island, Kingston, Rhode Island, United States; University of Rhode Island, Kingston, Rhode Island, United States; University of Rhode Island, Kingston, Rhode Island, United States; University of Rhode Island, Kingston, Rhode Island, United States; University of Rhode Island, Kingston, Rhode Island, United States

## Abstract

The quickly growing population of older adults in the United States combined with an increasingly digital society post-pandemic has illuminated the digital divide in recent years. The digital divide contributes to social isolation and therefore decreased quality of life in older adults, and intergenerational programming has demonstrated potential to bridge this equity gap and keep older adults involved and productive in their communities and larger society. To expand the literature and enhance the rigor of the research design in intergenerational programming, this study utilized a waitlist control design to strengthen outcomes from the University of Rhode Island Engaging Generations Cyber-Seniors Program on measures of technology use, digital competence, loneliness, social isolation, and quality of life. For this design, data was gathered at time 1 (6 weeks before the program), time 2 (2 weeks before the program), and time 3 (after participation in the program). Repeated measures ANOVA was used to examine changes across time points. Implementing a waitlist control design enabled us to examine natural changes that occurred in the measures compared to after program participation. Results showed that the program likely impacted tablet use, digital competence, and social isolation but that loneliness was impacted due to pre-program activities, such as the interview. These findings strengthen the previous research in understanding the specific aspects of social well-being that the program influences. This methodology was a practical way to utilize an already existing waitlist to enhance the rigor of evaluation research methods used.

